# Animal Wellness: The Power of Multiomics and Integrative Strategies: Multiomics in Improving Animal Health

**DOI:** 10.1155/2024/4125118

**Published:** 2024-10-24

**Authors:** Ratan Kumar Choudhary, Sunil Kumar B. V., Chandra Sekhar Mukhopadhyay, Neeraj Kashyap, Vishal Sharma, Nisha Singh, Sina Salajegheh Tazerji, Roozbeh Kalantari, Pouneh Hajipour, Yashpal Singh Malik

**Affiliations:** ^1^Department of Bioinformatics, Animal Stem Cells Laboratory, College of Animal Biotechnology, Guru Angad Dev Veterinary and Animal Sciences University, Ludhiana 141004, Punjab, India; ^2^Department of Animal Biotechnology, Proteomics & Metabolomics Lab, College of Animal Biotechnology, Guru Angad Dev Veterinary and Animal Sciences University, Ludhiana 141004, Punjab, India; ^3^Department of Bioinformatics, Genomics Lab, College of Animal Biotechnology, Guru Angad Dev Veterinary and Animal Sciences University, Ludhiana 141004, Punjab, India; ^4^Department of Animal Biotechnology, Reproductive Biotechnology Lab, College of Animal Biotechnology, Guru Angad Dev Veterinary and Animal Sciences University, Ludhiana 141004, Punjab, India; ^5^Department of Bioinformatics, College of Animal Biotechnology, Guru Angad Dev Veterinary and Animal Sciences University, Ludhiana 141004, Punjab, India; ^6^Department of Clinical Sciences, Faculty of Veterinary Medicine, Science and Research Branch, Islamic Azad University, Tehran, Iran; ^7^Department of Avian Diseases, Faculty of Veterinary Medicine, University of Tehran, Tehran, Iran; ^8^Department of Clinical Science, Faculty of Veterinary Medicine, Shahid Bahonar University of Kerman, Kerman, Iran; ^9^Department of Microbial and Environmental Biotechnology, College of Animal Biotechnology, Guru Angad Dev Veterinary and Animal Sciences University, Ludhiana 141004, Punjab, India

**Keywords:** animal health, livestock, multiomics

## Abstract

The livestock industry faces significant challenges, with disease outbreaks being a particularly devastating issue. These diseases can disrupt the food supply chain and the livelihoods of those involved in the sector. To address this, there is a growing need to enhance the health and well-being of livestock animals, ultimately improving their performance while minimizing their environmental impact. To tackle the considerable challenge posed by disease epidemics, multiomics approaches offer an excellent opportunity for scientists, breeders, and policymakers to gain a comprehensive understanding of animal biology, pathogens, and their genetic makeup. This understanding is crucial for enhancing the health of livestock animals. Multiomic approaches, including phenomics, genomics, epigenomics, metabolomics, proteomics, transcriptomics, microbiomics, and metaproteomics, are widely employed to assess and enhance animal health. High-throughput phenotypic data collection allows for the measurement of various fitness traits, both discrete and continuous, which, when mathematically combined, define the overall health and resilience of animals, including their ability to withstand diseases. Omics methods are routinely used to identify genes involved in host-pathogen interactions, assess fitness traits, and pinpoint animals with disease resistance. Genome-wide association studies (GWAS) help identify the genetic factors associated with health status, heat stress tolerance, disease resistance, and other health-related characteristics, including the estimation of breeding value. Furthermore, the interaction between hosts and pathogens, as observed through the assessment of host gut microbiota, plays a crucial role in shaping animal health and, consequently, their performance. Integrating and analyzing various heterogeneous datasets to gain deeper insights into biological systems is a challenging task that necessitates the use of innovative tools. Initiatives like MiBiOmics, which facilitate the visualization, analysis, integration, and exploration of multiomics data, are expected to improve prediction accuracy and identify robust biomarkers linked to animal health. In this review, we discuss the details of multiomics concerning the health and well-being of livestock animals.

## 1. Introduction

The multilayered characterization of the biological system extends our knowledge of an organism's functionality, in particular onset and progress of the disease, mechanisms of development, and in identifying disease-resistant animals. Multilayers of characterization include various omics approaches, including phenomics, genomics, transcriptomics, proteomics, microbial proteomics residing inside the host, and metabolomics. The study of important phenotypes and the identification of the associated molecular signatures have been facilitated by the analysis of single omics datasets [1] but are less reliable in predicting mechanisms underlying phenotypic variabilities [2]. Genomics identifies genetic variations in the genome associated with the disease, treatment response to the infection, underlying disease progress if genetic, and prognosis of the ailments. This has increased our knowledge of complex diseases through the successful identification of thousands of associated genetic variants through genome-wide association studies (GWAS) [3, 4]. Transcriptomics examines genome-wide RNA levels and determines which genes are differentially expressed under the disease conditions. The advent of sequencing techniques, like RNA-seq, offered the identification of novel genes and gene isoforms and showed the complexity of protein-coding transcripts. Dysregulation of noncoding RNA, microRNA, piwi-interacting RNA, and short nucleolar RNA have been implicated in various diseases. Proteomics identifies and quantifies peptide abundance, modification, and interaction. MS-based methods have revolutionized analyses of high-throughput protein sequencing in cells or body fluids. Other classical protein assay methods like the interaction between proteins, phage display method, and yeast two-hybrid assays are employed. The identified protein is validated by Western blot, while the interaction of DNA-protein is done by a ChIP-Seq assay.

Microbiomics is a fast-growing field where all microorganisms living as a community are investigated together. The presence of colonized microorganisms, including bacteria, viruses, and fungi on the skin, mucosal surface, and inside the gut, collectively called microbiota. Many studies have indicated that microbiota perturbation is associated with the onset of the disease. Microbiome profiling identifies the quantitative determination of microbiota taxa in healthy and diseased conditions. Likewise, metabolomics identifies multiple small molecules or metabolites like amino acids, fatty acids, and other cellular metabolites. Metabolites are the end products of the metabolic process occurring inside the cells and in response to an altered environment. The study of metabolites provides information about an individual's physiological and disease status.

Multiomics detects subtle shifts long before clinical symptoms appear. Imagine identifying metabolic imbalances or protein dysregulation before illness manifests. Early intervention becomes possible, potentially saving lives. Multiomics is not confined to research laboratories. It informs breeding programs, wildlife conservation, and livestock management. It empowers us to safeguard endangered species, optimize productivity, and promote animal welfare. However, challenges remain—data integration, computational complexity, and ethical considerations demand our attention. Armed with multiomics data, veterinarians, researchers, and conservationists can tailor interventions. Whether adjusting nutrition, designing targeted therapies, or predicting drug responses, personalized approaches enhance animal well-being [5, 6].

**Figure 1:**
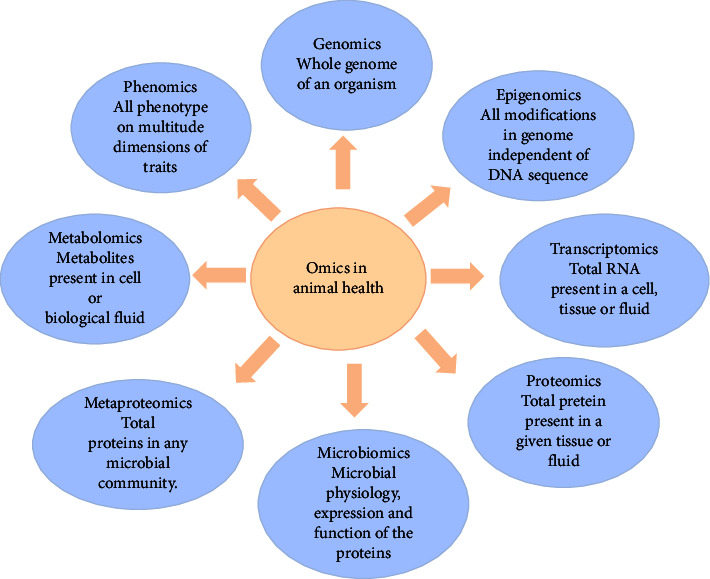
Overview of the utility of multiomics approaches in animal health; namely, phenomics, genomics, epigenomics transcriptomics, proteomics, microbiomics, and metaproteomics.

In this review, we discuss the detail of multiomics concerning the health and well-being of livestock animals. Each type of omics data provides a list of differences associated with the disease and may be limited to correlations and differences. We emphasized the integration of “omics” data to elucidate potential causative changes and understand the system biology in healthy and diseased conditions. This review explores the utility of multiomics approaches; namely, phenomics, genomics, epigenomics transcriptomics, proteomics, microbiomics, and metaproteomics (Figure 1). We further discussed how the integration of multiomics data could better understand biological systems better.

The multiomics approaches in animal health are discussed under subheads as.

## 2. Phenomics

In recent decades, technological progress has made it possible to conduct studies on an integrated-omics level, reducing the cost and time required to acquire information in genomics, transcriptomics, proteomics, and metabolomics.

Present-day genomics has progressed by leaps and bounds to emerge as a standard technology. However, a limitation has been evident: without identifying the causative mutations, increasing marker density or genome sequencing only adds marginal gains in genetic prediction accuracy [7, 8]. Further, an applied omics study always requires the omics data to relate to a phenotype of importance. Since phenotype recording still has a great potential for its improvement in scope and scale, reaching towards a high-throughput phenotyping, with the aid of advances in sensors, connectivity, and intelligent computing. This high-throughput phenotypic data recording is referred to as phenomics, defined as “the acquisition of high-dimensional phenotypic data on an organism-wide scale” [9].

The term phenomics is an all-encompassing word intended to be used for recording every possible phenotype on the multitude of traits dimensions. It thus is not finite or rigidly defined as the other omics disciplines. For example, the genomics data is simply the sequence arrangement of nucleotides in the genome. The high dimensionality arises mainly due to several polymorphisms of a few types. In contrast, the phenome consists of both discrete and continuous variations in a multitude of traits and a mathematical combination of theirs.

The technologies that allow the recording of phenotypes in animals are in increasing demand by the farmer community, most of which are related to farm management directly and to resource use efficiency, disease incidence, resilience, and animal welfare [10–12] in one way or another. Recent works indicate that phenomics is gaining momentum in the animal sciences [13–15]. Increasing milk production performance of dairy animals, for example, all aspects of dairy cattle production systems via, genetic improvement, on-farm management systems, and efficient utilization of resources, with an emphasis on welfare and health improvement of animals [16]. The progress around high-throughput phenotyping in livestock comes from either the possibility of measurement of traits that could not be recorded before or upscaling of existing attributes in a noninvasive and dense manner under routine production practices. A briefing about the newer traits and recent improvements in the phenotyping of more unique and classically recorded traits is presented hereafter.

### 2.1. Behavioural and Temperamental Traits

The behaviors of the animals at the individual as well as herd level are essential criteria in animals' utility and suitability for farming. A docile animal breed can be kept with little supervision in high stocking density while a comparative feral animal may be suitable for open ranches and guarding. It is possible to select to modify behavior since some behaviors, like mothering ability, are heritable [17]. The traits of behavioral importance can be measured with the help of sensors and cameras [18–20]. Bio-logging can be described as the tracking of identity, location, behavior or bio-physiological parameters of individual animals with equipment attached or implanted on them. Some more popular sensors include RFID tags, GPS tags, accelerometers, microphones, and video cameras. The number and types of such bio-loggers have exploded recently [21–23].

### 2.2. Physiological Parameters

The sensors are available to monitor body temperature, heart rate and muscular movements (with accelerometer), blood pressure, and oxygen saturation. Many of these sensors have been applied to wearable technologies for animals. The heart rate and temperature sensors can use algorithms to infer behavioral and other deviations such as stress and disease [24–26]. A wireless sensor–based system detects early avian influence outbreaks on a chicken farm. A prototype of a wearable wireless node detects accelerometer and increased temperature for a chicken and identifies highly pathogenic infection of avian influenza at an early stage [27].

### 2.3. Milk Production

In coordination with the radio frequency identification (RFID) animal identification system, the automated milking parlors can accurately record the daily milk yield of the animals. Milk autoanalyzers have been developed and improved to detect milk composition [28]. An automatic milking system with inline sensors for electrical conductivity, electrical resistance, and milk color can be used to detect mastitis—a grave condition affecting livestock globally [29–31]. Lactate dehydrogenase (LDH) in the milk can be measured by Herd Navigator from DeLaval Ltd., Cardiff, UK, by enzymatic reaction for inferring mastitis. The inline system was attempted to develop that measure LDH to determine post-treatment mammary *Staphylococcus aureus* infection status in dairy cows [32].

### 2.4. Reproduction

Many sensors such as pedometers, accelerometers, and microphones have been utilized to predict reproductive events such as heat and calving in farm animals. A lot of commercial solutions are also available in the market. Pedometers can continuously monitor cattle movements and predict the heat based on alterations in movement patterns. Increased numbers of steps in cattle were associated with the behavioral estrus period; thus, pedometers accurately detected ovulation time towards improving fertilization rates [33]. Advances in the accuracy of estrus prediction and calving developed a multifunctional detection system in cattle by combining accelerometers and ultra-wideband sensors [34].

### 2.5. Growth and Body Confirmation

Weighing bridges in line with milking parlors or at passages can automatically log the body weight of the animals, tracking their development. Depth-sensing cameras in a set configuration can successfully record various body measurement parameters, giving an alternative to animal growth monitoring [35, 36].

These sensors have been adapted to wearable technologies for the animals, provided with the options to connect wirelessly to the data loggers and cloud database. Through continuous monitoring of the animals, these sensors, which primarily aim at precision livestock farming, produce a considerable amount of phenomic data on the animals. It is estimated that the market for wearable animal sensors will increase more than 2.5 times from 2017 to 2027 [37]. Artificial intelligence (AI) algorithms have been extensively applied to utilize this big phenomics data to analyze, interpret, and abstract into simple and valuable information. The precision livestock farming concept heavily relies on managing the rearing conditions of livestock based on the phenome information. This helps to improve production efficiency and reduce physical labor and labor cost. Further, a high-throughput phenome data are the biggest asset to associate it with other multiomics data to identify the bio-molecular mechanisms of animal production and health.

## 3. Genomics

An organism's genome refers to the whole hereditary information (including both the genes and the noncoding sequences) and consists of DNA (except for some RNA viruses). Animal's genome carries the typical connotation of chromosomal DNA besides mitochondria contain genes; these genes are not considered part of the genome but are termed “mitochondrial genome.” While the term genomics was coined recently, in 1986, scientist Tom Roderick worked in Jackson Laboratory. “Genomics” is the branch of biological science that revolves around studying evolution, structure, function, genetic interactions, genome mapping, genome annotation, etc. Genomics, mainly concerned with sequencing the genomes of various organisms, has heralded the era of functional genomics (different gene expression patterns), encompassing the techniques of microarrays and bioinformatics. With the abundance of massive biological datasets, computational studies have become one of the most important means of biological discovery. Computational genomics aims at deciphering the encrypted messages at the genomic level through bio-computational analysis to explore the treasure of knowledge underlying the principles of central dogma and reverse dogma.

Various branches of “omics” (viz., Genomics, proteomics, transcriptomics, and informatics) have found their applications to unravel the hidden treasure of systems biology information. To handle so much of primary data, plenty of software is required. In-depth knowledge of that software, its uses and applicability should be learned to derive meaningful conclusions from the experiments conducted on genomics and proteomics. In a broader perspective, the study of genomics has helped to understand the following:

• Genetic marker: marker-assisted selection (MAS) through black-box approach
• Genomic selection: extrapolating the concept of MAS to genome-wide distributed markers
• Genome-wide association study: to identify the underlying causal variations at the genomic level
• Evolutionary biology: genome and species evolution
• Cellular signaling networks: gene-gene interaction
• Genome mapping

In this section of the current review, the applications of genome research for animal science are discussed.

### 3.1. Genetic Markers

These are a kind of molecular markers that are stably inherited and remain unaffected by age and sex and are used to screen and indirectly select the individuals carrying the alleles/haplotypes of interest. Since 1970, several genetic markers have been discovered, some of which have been extensively used for the selection of breeding individuals, breed identification, parentage determination, and evolutionary studies. A popular method of animal selection for breeding purposes is MAS, which uses a set of genetic markers.

### 3.2. MAS: A “Black Box” Approach

Quantitative trait loci (QTLs) refer to the specific genomic location that houses the genes controlling a quantitative trait. The set of genes of a QTL may be spanned over multiple chromosomes, although these contribute towards a common trait (which is generally a complex trait as well). Molecular markers enable us to tag these loci and identify the major genes contributing to the trait of interest. In MAS, we classify the genes in a QTL into two types, major genes and minor genes. The effect of major genes is considerably high. So, these genes are targeted for the selection of individuals. Such genes/loci are genetically mapped by molecular markers viz., SNPs that are linked or associated with a QTL. Such association can be estimated by linkage disequilibrium (LD) studies. This selection strategy using MAS is popularly compared with the “Black Box” approach because the input of each locus towards the complex trait is not precisely measured. Thus, MAS is an indirect measure of the selection of breeding individuals. The molecular markers are utilized as a “genetic determinant” of the trait of interest. MAS is called an “indirect measure” since an individual is selected based on the marker associated with the desired phenotype, however, not by the actual gene that is governing the trait. The set of markers (for MAS) is first identified in a training population and then the markers are validated in a different set of the test population. MAS is preferred for traits that are difficult to measure or traits that are expressed later in life or after the animal's death (like slaughter traits) and for sex-limited traits [38].

### 3.3. Genomic Selection (GS)

GS is a selection decision based on the calculated genomic breeding expected values (GEBV). The GEBV's are obtained as the sum of the marker-effects for all the qualifying genetic markers across the entire genome. Thus, it captures all the QTLs contributing to a trait's variation. Genomic selection is the pan-genomic method of MAS. Genomic selection attempts to identify the genetic markers distributed genome-wide [39], where each quantitative trait locus is required to be in LD with at least one of those genetic markers (viz., SNPs). Haley and Visscher first pioneered the use of the term “genomic selection” at Armidale World Congress on Genetics Applied to Livestock Production (WCGALP) in 1998 [40]. Meuwissen et al. published the first simulation study on GS [41]. The basic assumption behind GS is that the summation of the marker effects yields the GEBV. These dense genetic markers are about 1 cM apart and are in LD with the QTLs. After 3 years of publishing this paper (i.e., 2004 onwards), some articles were published reporting the simulation studies on genomic selection [42, 43].

Genomic selection is conducted in two significant steps:

i. Training population: The markers that are exhibiting LD with the QTL are accounted for in the marker effects in the training population. This population harbors the animals with phenotypic and genotypic information (marker genotype information of each animal). The marker-effects are summed up to calculate the molecular breeding value for each of the contributing loci based on the prediction equation developed [40, 44].
ii. Test or validation population: We get the marker effects from the training population. The results are then applied to predict the molecular breeding values of the individuals of the validation (or test) population feeding the prediction equation with the SNP/genotypes. The reliability of the prediction in the test population is verified with the observed phenotypic record of this group of animals.

### 3.4. GWAS

It tests all the pan-genomic-SNPs for their association with the trait of interest. GWAS is a “noncandidate-specific” approach, which means the GWAS method identifies the set of SNPs associated with a trait of interest (high or low productivity, disease resistance or susceptibility, etc.) between “case” and “control” individuals. GWAS have identified the QTLs dictating complex diseases in humans and mice [3]. The first GWAS was reported on human-patients with a condition called “age-related macular degeneration” in humans by Klein et al. [4].

## 4. Epigenomics

In recent years, continued technological advances in genomics have helped in the selection of animals at an early age for desirable traits of production, disease resistance, and stress tolerance [45, 46]. Genomics has helped in understanding the regulatory and general role of the noncoding and coding sequence in the DNA underlying the production and health traits and thus improvement in the animal breeds for the desirable trait [47, 48]. Despite these advances, optimal improvements in animal health and production have not been achieved due to several reasons. Thus, it becomes imperative to look beyond the DNA sequences as there are other factors that play a role in determining the function of a gene at a particular stage of the cell.

**Figure 2:**
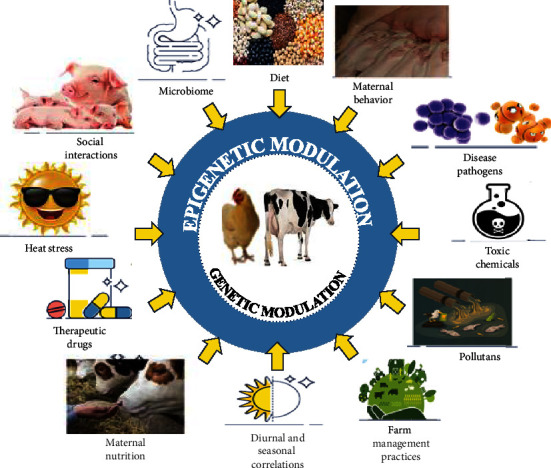
Factors leading to differential epigenetic modifications in the genome [50].

Epigenomics is the field of science that studies epigenetic modifications (chemical tags) that mark the genome. The expression of the gene in a cell and its function in a cell are not dependent entirely on the sequence of that gene in the genome. The environmental factors, nutrition, physiological, and health status of an organism also change the expression of the gene. It has always fascinated researchers how the expression of a gene changes across different conditions though the DNA sequence remains the same. Some of the answers to these questions can be found by studying epigenetics because the gene expression, function, and impact on the other genes depend on the epigenetic status like DNA methylation, acetylation, phosphorylation, histone modifications of the gene, and associated proteins. Epigenetics studies the heritable molecular modifications in the gene that regulate the gene expression in the cell independent of the primary DNA sequence of the gene [49]. Epigenomics studies the impact of factors that bring epigenetic changes across all the genes or genomes of a cell, tissue, or whole organism (Figure 2). Epigenomics is different from epigenetics in the aspect that epigenetics focuses on the mechanisms involved in the changes of gene expression due to the epigenetic changes in the genes that do not alter the DNA sequences, while epigenomics studies the effect of all epigenetic changes in the genome of an organism. Non-coding RNA regulations, chromatin remodeling, histone modifications, and DNA methylation involve different epigenetic modifications involved in regulating cell function.

### 4.1. Epigenomics and Animal Reproduction

Dynamic epigenetic modifications occur during the development of an organism. Various researchers have reviewed the vital role of different epigenetic changes and their impact on livestock's embryonic and placental developmental phases [51–53]. DNMT3A and DNMT3B establish DNA methylation patterns in response to environmental challenges and during embryonic development [54, 55]. DNA methylation pattern during embryonic development impacts various metabolic and differentiation processes, which affect embryo vitality and fetus development [56–58]. Heat stress regulates epigenetic modifications like DNA methylation, histone modifications, and DNA hydroxymethylation in an organism, and fertility and embryonic development in cows have been reported to be affected by it [59–62]. Poor performance of the offspring born to a mother exposed to different stressors like heat stress, negative energy balance, and metabolic disorders have been partly explained by the changes in the differential DNA methylation pattern across different tissues [63–65]. Fertility and related traits are influenced by histone modifications [66, 67] and abnormal DNA methylation pattern in sperm [68–70] in the bull. Bull fertility [71], fertilization, spermatogenesis, and methylated regions associated with reproduction traits [72] may be predicted by candidate genes identified through whole-genome bisulfite sequence (WGBS) of low- and high-fertility bull semen. Sperm fertility and functionality have been associated with differential methylation patterns in the genes related to repetitive element activities and chromatin remodeling in pericentric regions [73]. Age, heat, and oxidative stress also change the methylation of the sperm genome [74–77].

DNA methylation changes during different embryonic stages in broilers' genes of muscle development play an essential role in the development of embryonic muscle [78]. During the mid-stages of gestation in pigs, the DNA methylation analysis in the brain tissues of pigs revealed a decrease in the expression of DNMT1, DNMT3A, and DNMT3B genes, suggesting that piglet brain development is influenced by DNA methylation [79]. Differential methylation analysis of weakly and strongly inbred chickens has revealed that the reproductive system's suppressed development may be due to the DNA's methylated regions and genes in the chicken [80]. Transcriptional N6-methyladenosine (m6A) profiling of the granulose cells of pigs suggested that high m6A modifications may be associated with folliculogenesis and steroidogenesis in pigs [57].

### 4.2. Epigenomics in Animal Production

Differential epigenetic profiling of the somatic cells and tissues, including the mammary glands, brain, muscles, liver, and small intestine of the animals, suggested that epigenetic changes impact growth and productivity in the animals [78, 79, 81, 82]. DNA methylation of different genes or their promoters regulates the differentiation of adipocytes in bovine [83], tooth development in pigs [84], changes in gene expression in muscles, and genes involved in response to heat stress in pigs [85]. Differential DNA methylation of *Longissimus dorsi* muscles in sheep and pig has revealed the role of DNA methylation in muscle development [86, 87]. Differential methylation has also been associated with muscle development and meat quality in beef [88, 89] and milk quality [90]. Likewise, meat quality gene differential DNA methylation in juvenile layers and later laying period hens identified the role of epigenetics in intramuscular fat deposition [91]. DNA methylation has also been shown to be associated with boar taint in pigs [90], goat milk traits [92, 93], the transformation of fur with unique characteristics and development of wool fiber [94], and the genetic stability of wool traits between different cashmere goat generations [95].

### 4.3. Epigenomics in Animal Health

Epigenetic modifications help animals in combating environmental stressors and maintain their health. The effect of heat stressors on epigenetic changes and, after that, on animal reproduction and embryonic growth and development has already been discussed. Hypoxic stress, due to low environmental oxygen at high altitudes, is another important environmental stressor that affects the growth of animals, especially porcine growth, at high altitudes. Differential DNA methylation studies in pigs have shown the role of methylation of the HIF-1 gene [96], SIN3A, and CACNG6 in adaption at high and low altitudes [97]. These epigenetic modifications confer better adaptability to hypoxic stress.

Most of the disease resistance traits have low heritability. Therefore, environmental factors and epigenetic changes conferring the disease resistance become more critical. In recent years, regulatory mechanisms involved in developing disease and disease progression symptoms have caught the attention of researchers leading them to explore the potential role of epigenetic modifications on animal health and disease resistance traits. Epigenetic alterations in different genes help regulate disease symptoms and thus assist in providing tolerance to pathogens. Epigenetic changes have been associated with developing inflammation in tissues [98]. Alterations in DNA methylation in different genes are associated to the formation of clinical and acute mastitis [48, 99]. In chickens, the ACC and MTTP gene promoter region's DNA methylation status has also been associated with the fatty liver syndrome [100]. Post-transcriptional RNA modification by miRNA, another epigenetic modification, is of great importance in diagnosing the disease status of animals. For example, upregulation of bta-mir-223 and bta-mir-21-3p miRNA was observed in the *S. aureus* infected quarters of the udder in the cow by Fang et al. [101]. miRNAs and their association with mastitis and other diseases of livestock have been reviewed in detail by different researchers [102, 103].

## 5. Transcriptomics

Transcriptomics investigates the gene expression level in a particular cell and in a specific state, such as in diseased conditions. Up- and downregulation of these transcripts in specific conditions changes specific metabolite and protein levels, leading to changes in the animal's phenotype [104]. Regulation of gene expression across various tissues and other biological states determines the mechanisms underlying phenotypic variation. Understanding mechanistic regulation of such changes is essential to incorporate molecular phenotypes into genetic improvement programs. Besides DE gene analyses, transcriptomic studies analyze gene-gene interactions by using a network approach for the detection of clusters of co-regulated genes. Next-generation sequencing approach identifies novel genes and characterizes spatial and temporal changes in gene expression or phenotypes. Phenotypes result from the molecular cascade that proceeds from DNA to RNA to protein and metabolic substrates. The techniques used to characterize transcriptome profile include microarray technology and next-gen RNA sequencing (RNA-seq). Detecting new genetic variants and transcripts is facilitated by the shift to RNA-seq data from microarray expression data. RNA-Seq provides further information on the ability to quantify and identify haplotype-specific expression, allele-specific expression, exon-specific expression, and isoforms [104]. Usage of genomic and transcriptomic technologies appeared to be instrumental in understanding the origin, development, and pathogenesis of many cancer types in canines [105].

Blood transcriptome was a potential diagnostic tool for identifying biomarkers of bovine respiratory disease (BRD) infection and was helpful in understanding virus infection and disease status in cattle. The transcriptome profile of whole blood showed a distinct antimicrobial peptide-driven host immune response that was occurring in the cattle affected with BRD [106]. The authors concluded that understanding the role of genes in establishing BRD in beef cattle may have potential therapeutic applications. Similar studies using BRD-susceptible and BRD-resistant calves revealed DE genes and molecular processes involving microbial killing. Production of specialized pro-resolving mediators (SPMs) and endogenous metabolism of angiotensinogen was increased in BRD immune calves, which may include microbial killing [107], and key genes (IFI6, IFIT3, ISG15, MX1, and OAS2) were identified as biomarkers to predict and recognize BRD sick cattle at entry [108].

In order to detect gene-based early detection of biomarkers of bovine mastitis before its severity, transcriptomic data of previously published studies were analyzed in silico. Data of four different areas of the mammary glands in control animals vs. animals 24 h after infection with *E. coli* were studied [109]. Four different regions of the mammary gland were Furstenberg's rosette, lobuloalveolar, gland cistern, and teat cistern and the number of DE genes from these regions was 636, 577, 597, and 453, respectively, of which, 101 genes overlapped. These genes were connected with eight pathways associated with immune responses, including chemokine signaling pathways (HCK, CCR1, NCF1, IL8, and PTK2) and NOD-like receptor signaling pathways (PYDC1, IL1B, IL18, and IL8) [109]. Furthermore, in silico analyses of 3D structure docking models of DE gene proteins identified their active sites, which could be utilized in finding appropriate binding ligands for making or selecting the suitable treatment or feed for animals infected with *E. coli* at an early stage.

With the advancement of transcriptomic sequencing, it is now possible to sequence at a single cell level, called single-cell RNA sequencing (scRNA-seq). scRNA-seq data of 5064 and 1372 individual ruminal epithelial cells from two Holstein calf tissues before and after weaning identified six distinct clusters of genes. Analyzing clusters of genes of ruminal epithelial cells under the cell cycle, pseudotime trajectory, regulatory network, weighted gene co-expression network, and gene ontology revealed an association with animal production and body conformation traits [110]. The study of scRNA-seq may open discoveries of cell or tissue types in determining complex traits.

## 6. Proteomics

Proteomics is the study of the proteome, i.e., the complete list of proteins in fluid or tissue. The significance of proteomics has been overwhelmingly recognized in recent years, primarily as an approach to improve health, but its full application in animal production and health is yet to be achieved. However, detecting probable biomarkers of various maladies of diverse animal species and explaining the physiological and pathophysiological causes of challenges using quantitative proteomics are now possible with the establishment of a helpful pipeline of proteomics methodology [111].

### 6.1. Proteomics in Animal Health and Production

Maintaining the health, growth, and fertility of animals is the first step in farm animal management, if we are to produce animal products at the highest quality. Increased demand for food of animal origin has led to the adoption of more stringent manage mental conditions that have further induced stress in animals [111, 112]. Stress renders farm animals vulnerable to metabolic and infectious diseases and other maladies related to production [111, 113]. Proteomic examinations of bovine mastitis have mostly been carried out on bovine milk due to the ease of sample collection using approaches like 2D electrophoresis followed by MALDI-TOF (matrix-assisted laser desorption/ionization time-of-flight)/MS and liquid chromatography associated with LC-MS/MS (tandem mass spectrometry) [114]. Various research groups have identified differentially expressed markers in milk and serum in the case of bovine mastitis using proteomic approaches. Marked decrease in *α*-lactoglobulin, *β*-lactoglobulin and caseins simultaneous with increase in serum transferrin and albumin in the whey collected from cows with mastitis [115] and an increased expression of serum amyloid A, heat shock protein 70, cathelicidin-I, and apolipoprotein A-I (apo A-I) in bovine mastitic milk [116] have been reported using proteomics approaches [114]. Similarly, proteomics has also been applied to the study of host-pathogen interaction and detection of novel vaccine biomarkers. For example, adaptation signatures have been placed in the case of avian flu in the human host [117], and responses to the virus have also been characterized in chicken [118]. Proteomics has already been applied to study the pathogenesis of herpes viruses [119], with a particular focus on Marek's disease [120].

In India, animal science is overseen by the State Agriculture University system and the aegis of ICAR (Indian Council of Agricultural Research). In 2008, ICAR funded the National Agriculture Innovation Project (NIAP), which provided funding support for the start of Animal proteomics. Proteomics research is also going on in ICAR-National Dairy Research Institute (ICAR-NDRI), Karnal, India, where facilities like 2-DE, MS are instituted. Also, similar facilities are available at New Delhi's ICAR-Indian Agriculture Research Institute (ICAR-IARI).

Animal production systems have also benefited from domestic animal proteomics. These studies include the characterization of bovine milk proteome and differentiation between breeds [121, 122], lactating mammary epithelial cells in water buffalo [123, 124], and cattle, or the establishment of protein biomarkers for estrus detection [125], early pregnancy diagnosis [126], and male fertility [127, 128].

### 6.2. Proteomics in Animal Reproduction

Reproductive processes at a molecular level are complex phenomena that occur by interaction of multiple proteins. To understand the reproductive mechanisms, proteomics can identify female- and male-specific reproductive proteins [129]. Present animal breeding procedures are totally linked with assisted reproduction technologies (ART) [130]. At the beginning of the last century, animal reproduction techniques have progressed from the addition of artificial insemination and now a variety of techniques are regularly used such as in vitro maturation of male and female gametes, semen preservation, embryo transfer, and in vitro embryo production [131–133]. In traditional farm animals, advanced reproductive technologies are used, which involve genetic selection methods for better reproductive traits and production [134].

The level of protein expression, their localization, isoforms made by alternate splicing and post-translational modifications along with functional protein interactions with biomolecules as well as resulting signaling cascades, all these things cannot be detected at the nucleotide level [135]. Several seminal-specific proteins have been identified by proteomic research, and new reports have shed more light on their role in male fertility. These proteins might help distinguish between infertility and fertility clinically if more research is done to validate them. For a more accurate clinical diagnosis and course of treatment for male infertility, proteomic studies may aid in the development of new methods for identifying novel biomarkers. The identification of novel pathway and molecular mechanisms in male infertility could significantly contribute to the development of novel therapeutic strategies in this field [136]. Comparative studies between high-fertility and low-fertility animals reveal differences in protein profiles. These variations may correlate with reproductive efficiency [137]. Also, proteomics helps unravel the intricate interactions between pathogens (such as bacteria or viruses) and reproductive tissues. By studying protein changes during infection, we identify disease-specific markers [138]. Few studies have reported how comparative proteomics invented pig oocyte putative quality markers by quantitative tandem mass spectrometry after differential labeling of proteins by ExacTags. Transforming acidic coiled-coil containing protein 3 (TACC3) expressions in mouse immature GV-stage oocytes was shown by proteomic studies of various stages of oocyte maturation using tandem mass spectrometry [139].

Moreover, a number of profuse molecular chaperones and heat shock proteins have been observed and their localization determined on the plasma membrane of mature mouse oocytes in the MII stage [140]. This study indicates that in addition to their involvement in their usual actions, including oocyte environmental response by regulating defense mechanisms (apoptosis) and protein folding, molecular chaperones and heat shock proteins may also play a central role in oocyte maturation [141].

## 7. Microbiomics (Microbial Proteomics)

Microbial infection is a major reason for death all over the world. Drug-resistant microbes originate the majority of the infections, and this may result in a delay in delivering microbiologically adequate treatment [142, 143]. Therefore, to defeat microbial infections, it is important to understand microbial physiologies, defense systems, survival strategies, and infection. Microbial proteomics is the field that conveys hints in antimicrobial diagnosis and therapy in clinical settings and imparts a thorough knowledge of expression and function of the proteins playing roles microbial infections and physiology [144, 145]. Microbial proteomics facilitates the identification of proteins associated with microbial activity and host-pathogen interactions and antimicrobial resistance mechanisms. Various techniques like gel-free methods and 2-D gel-based methods in combination with MALDI TOF-LC-MS/MS can be used to confirm the activity of infectious microbes [146].

Various proteomic techniques are used to study the interactions between the host and the pathogen. Some such methods are yeast 2-hybrid, chemical cross-linking mass spectrometry, affinity purification-mass spectrometry, protein microarray-based technologies, protein correlation profiling, and proximity-dependent labeling strategies-mass spectrometry [147].

Bacterial identification, nucleic acid-targeting, and susceptibility testing benefit greatly from MALDI-TOF MS in clinical microbiology [148]. Bacterial infections in blood infections, the urinary tract, and other clinical samples can be detected in early stages of the infection using the MALDI-TOF MS technique. Microorganism classification, identification, and characterization employ MS-based proteomic techniques in their clinical diagnostic procedures. By assessing proteins affected by the disease, it is possible to screen and diagnose cancer at its early stages using proteomic tools [149, 150]. The diagnosis of tuberculosis in clinical isolates has been achieved using proteomic technology to estimate the rRv3874 and rRv336Z pathological proteins in vitro [151, 152]. Resistance to treatment and variations in insensitivity can be addressed by identifying cancer cells that are differentially expressed using proteomic methods [153].

## 8. Metaproteomics

Various proteins vary in their existence and abundance in microbial colonies. The study of such variations falls within the scope of metaproteomics. Using a suitable protein sequence database, proteins can be assigned to individual species or higher groups, as well as estimate the interactions and functional roles of each member in the community. Studies on pure cultures of various organisms have demonstrated that the activity and connection of a protein can be understood by assessing its abundance under a given condition. For instance, the environmental energy sources used for fixing carbon by the symbionts of a marine worm could be identified using a proteomics-based approach [154, 155].

Most metaproteomics studies use shotgun proteomics to evaluate bacterial proteins that may be affected by sensitivity and complexity issues, e.g., it may be difficult to identify proteins of lower abundance. However, the complexity and the dynamic range problem may be addressed by giving library-based methods a broader coverage for protein/peptide identification through applying data-independent acquisition (DIA) and other new spectral library-based methods [156–160].

Recently, various software tools have been developed specifically for metaproteomic data analysis. MetaLab is the software that uses spectral clustering to upgrade the speed of peptide detection [161]. Through creating a reference database by reading protein sequences from metagenomics sequence data using de Bruijn graph assembly, other studies have achieved better protein identification in metaproteomics [155, 159, 162]. Metaproteomics data can also be analyzed by MetaPro-IQ, which is suitable for fecal samples [163].

Identification of a protein is challenging if the taxonomic composition is not known because of the protein databases are missing some protein entries. For instance, the proteins of merely 1,240,268 (status, till 29 March 2021) species are included in the TrEMBL/UniProt database whereas it is estimated that the number of microbial species on Earth is up to one trillion [164]. Therefore, a little variation in the protein sequence among related microorganisms introduces a hurdle to the identification of the peptides for the target protein because it results in completely different tryptic peptides [155].

### 8.1. Isobaric Tagging for Relative and Absolute Quantification (iTRAQ)

iTRAQ is a major advancement in quantitative proteomics and toxicogenomics. As compared to other methods like Isotope-coded affinity tags (ICAT), differential gel electrophoresis (DIGE), and 2DE, iTRAQ provides higher sensitivity, and better quantitative reproducibility [165]. iTRAQ is a peptide labeling method [166] and involves the covalent labeling of N-terminus and side chain amines of peptides from protein digestions with tags of different masses [167]. iTRAQ can detect up to eight biological samples simultaneously using four or eight isobaric tags [166, 168]. There are two reagents; 4-plex and 8-plex that are commonly used to label all peptides from various samples or treatments. These samples are further pooled, fractionated using nano-liquid chromatography, and then evaluated by tandem mass spectrometry (MS/MS) [167]. Each reagent in the 4-plex and 8-plex versions contains a reactive peptide group along with an isobaric tag which comprises a balanced group and a reporter group [165].

iTRAQ technique is now widely used in various types of cancer therapy as well as used in measuring global protein content from malignant and nonmalignant tissues [167, 169]. This method is also used in the evaluation of blood transfusion biomarkers [170]. iTRAQ process is also useful in the functional quantitation of mitochondrial protein phosphorylation [171]. The iTRAQ method has been widely applied to investigate the proteomes of cattle, chickens, and pigs.

## 9. Metabolome

Metabolomics is a new field of science which study all the metabolites present in the cell and biological fluid of an organism at a particular stage. Metabolites are also referred as canaries of the genome [171] as they can provide information about the problems in the genome, transcriptome, or proteome. The term metabonomics is also used interchangeably with metabolomics though they have different meanings. Metabolomics aims to catalog and identify total metabolites present in biological fluids in diverse conditions, while metabonomics concentrates on studying the profiling of the metabolites under other conditions [171]. Metabolites are the end products of the various mechanisms occurring inside the cells and in response to the outside environment. The study of metabolites provides information about the physiological and disease status of an individual and changes occurring inside the cell in response to environment stimuli. Hence, metabolomics provides more than useful information about the phenotype [172, 173]. Metabolomics complements genomics, transcriptomics, and proteomics studies and has found applications in different fields [174] like toxicology [175], nutrition science [176], environmental science [177], disease markers [178], food science [179], and systems biology [180].

Due to the recent advances in the science and tools available to study metabolites, metabolomics is gaining much more attention from the last few years. However, the application of metabolomics in the livestock industry is much less widely used when compared to agriculture science [181]. This is surprising considering the fact that the metabolomics study can be used noninvasively on some of the biological fluids which may provide information about the phenotype [182–184].

The main tools to study metabolomics are MS and Nuclear Magnetic Resonance (NMR) combined with chromatography. There are different approaches for the analysis of the metabolites detected by various tools [185]. Targeted metabolite analysis focusses on some specific metabolites or a small set of molecules. Metabolic profiling studies the metabolites of particular pathways to get information about the physiological stage of the organism. Metabolite fingerprinting studies global metabolite profiling without any prior knowledge in order to identify some novel metabolites which may be associated as fingerprints of breed, species, and physiological or pathological status of an organism.

Metabolomics has been used to study chemical profiles and dynamic changes occurring in the milk under different conditions. Metabolites present in the milk may represent a biomarker for disease, lactation stage, nutrition status of the animal, the stress in the animal, and breed or species characteristic. Different researchers used metabolomics studies to reveal that milk from other breeds have the varying concentration of metabolites in their milk. Milk from Brown Swiss cattle has higher concentration of *α*-aminobutyric acid and a lower level of ornithine in comparison to Simmental cows [185]. Similarly, Yang et al. identified a differential concentration of 68 metabolites in the milk of cows from Holstein and Jersey breeds [186]. In general, choline and citrate have been suggested as candidate biomarkers to differentiate between the raw milk of Jersey and Holstein cattle (reviewed by [183]). Metabolite composition of milk have also been used to identify the biomarkers to distinguish milk from different species viz., cow and goat [186], cow and sheep [187], cow and buffalo [188, 189], cow and yak [186], cow and nonruminants [186]. Similarly, milk and colostrum of same animal shows a different concentration of metabolites in their milk. Li et al. observed that 11 free fatty acids (FFA) were significantly different between milk and colostrum of donkeys [190].

Similarly, milk from different lactation stages has different metabolite concentration in cattle [191, 192]. Milk metabolite concentration also shows variation in different seasons [193], and fodder given to the animals also affects the metabolites of milk [194]. Metabolomics study of milk has also provided information about the quality of milk-coagulation property [195].

Metabolomics study has also helped in the identification of biomarkers of different diseases. As drop in milk yield is one of the early symptoms of diseases in animals thus, changes in the metabolite concentration in the milk will also occur in disease conditions. In ketosis, a metabolic disorder, ketone bodies in milk, blood and urine have been accepted as a diagnostic biomarker [91]. Klein et al. suggested that in ketosis, the ratio of glycerophosphocholine to phosphocholine (less than 2.5) in the milk can be used to predict ketosis in cattle [196]. Similarly, in mastitis different metabolites in the milk have been suggested as biomarkers for the early diagnosis in combination with the changes in the somatic cell count (reviewed by [195, 197, 198]). Similarly, metabolomics studies of other biological fluids like the serum, plasma, saliva, and tissue have been used to identify markers for different diseases, viz., cancer, metritis, viral and bacterial infections, and epilepsy in other animals. Zhang et al. detected alterations in the multiple metabolites in preketotic, ketotic, and postketotic cow urine. Metabolites also serve as biomarkers for early diagnosis of cancer [199]. The citric acid in urine, blood and tissue has been proposed as a marker of prostate cancer in dogs [200]. Tsamouri et al. identified some metabolites which may serve as a biomarker for urothelial cancer in dogs [184].

Metabolomics study of tissue, saliva, plasma, rumen fluid, and feces has been used for the identification of differential metabolite changes under heat stress [199, 201–204]. Heat stress causes changes in the microbiota of the gut and vagina and metabolic profiles in animals [205]. Thus, metabolomics approaches may help identify potential biomarkers for detecting heat stress in animals. Different metabolite biomarkers of animal reproduction have also been studied in ovarian follicular fluid of cows [206], and semen of bulls [207, 208], and pigs [182]. But still, a conclusive study is required to associate metabolites with animal reproduction.

## 10. Integration of Multiomics Data

Multiomics characterization facilitates the investigations of interactions and associations across and within omics data. It offers a characterization of biological systems, like high disease-resistant animals, and improved performance, and knowledge on how organisms function. The identification of the molecular signatures the phenotypes are associated with has been facilitated by single omics datasets [182] but is less reliable in predicting underlying phenotypic variabilities [2]. Multiomics information helps to understand the flow of information that underlies the disease and delineate key players—genes, proteins, metabolites, and others [209, 210]. MiBiOmics is an online tool that uses a graphical user interface (GUI) to provide robust signatures of high-dimensional datasets. MiBiOmics takes a guided and intuitive approach to allow biologists to analyze multiomics data. Considering the challenges of a multiplicity of techniques, MiBiOmics provides pipelines for exploratory ordination techniques, correlation networks, dimensional reduction of the data, and association to contextual parameters [210].

## 11. Conclusions

The livestock industry grapples with substantial challenges, particularly disease outbreaks, which not only disrupt the food supply chain but also jeopardize the livelihoods of those involved in the sector. Enhancing the health and well-being of livestock animals is imperative for improving performance while minimizing environmental impact. Phenome and genome-based analyses may help in manage mental decisions to maximize the selection of animals with better health and disease resistance. Additionally, optimization of animal production through precision breeding and management is likely to determine the best phenotype of the animal. Transcriptome analysis in domesticated species may complement GWAS of complex disease traits with economic importance; however, such studies are still challenging in nonmodeled domestic livestock animals due to the lack of quality genome assemblies. The established proteomic pipeline, which starts with sample preparation followed by mass spectrometry, statistics, bioinformatics, and finally, biochemical analyses, can be further integrated with many areas of research, revealing novel markers that can be exploited for research on animal production and health. Extensive collaboration is necessary for a proteomic investigation of this scale between relevant disciplines, viz., biotechnology, bioinformatics, statistics, veterinary medicine, and animal production, to make the full potential of the omic sciences and the recent advances in omic technology accessible to researchers. Joint efforts accelerate scientific progress. Metabolomics study of different biological fluids like milk, plasma, serum, saliva, rumen fluid, tissue, and microbiota of animals has provided novel insights in animal science in various fields varying from milk composition, quality, and its commercial properties, heat stress detection, and disease diagnosis. Livestock health directly impacts food security and economic stability. Metabolomics study is still less widely used in animal science, and its integration with proteomics and transcriptomics may help further advance animal science. By leveraging multiomics strategies, stakeholders in the livestock industry can make informed decisions to enhance animal health, bolster disease resistance, and ultimately ensure sustainable productivity and welfare.

## Data Availability

The data used to support the findings of this study are included within the article.

## Nomenclature

GWAS: Genome-wide association studies
RFID: Radio frequency identification
LDH: Lactate dehydrogenase
AI: Artificial intelligence
MAS: Marker-assisted selection
QTL: Quantitative trait loci
LD: Linkage disequilibrium
GS: Genomic selection
GEBV: Genomic breeding expected values
WCGALP: World congress on genetics applied to livestock production
WGBS: Whole-genome bisulfite sequence
RNA-seq: RNA sequencing
BRD: Bovine respiratory disease
SPMs: Specialized pro-resolving mediators
scRNA-seq: Single-cell RNA sequencing
apo A-I: Apolipoprotein A-I
NIAP: National agriculture innovation project
ICAR-NDRI: ICAR-National Dairy Research Institute
ICAR-IARI: ICAR-Indian Agriculture Research Institute
ART: Assisted reproduction technologies
TACC3: Transforming acidic coiled-coil containing protein 3
DIA: Data-independent acquisition
iTRAQ: Isobaric tagging for relative and absolute quantification
ICAT: Isotope-coded affinity tags
DIGE: Differential gel electrophoresis
MS/MS: Mass spectrometry
NMR: Nuclear magnetic resonance
FFA: Free fatty acids
GUI: Graphical user interface
